# The Battle Within: A Qualitative Meta-Synthesis of the Experience of the Eating Disorder Voice

**DOI:** 10.3390/healthcare12222306

**Published:** 2024-11-18

**Authors:** Panagiota Tragantzopoulou, Christos Mouratidis, Konstantina Paitaridou, Vaitsa Giannouli

**Affiliations:** 1 School of Social Sciences, University of Westminster, 115 New Cavendish St., London W1W 6UW, UK; 2 School of Psychology, Mediterranean College, 54625 Thessaloniki, Greece; chi.mouratidis@mc-class.gr (C.M.); k.paitaridou@mc-class.gr (K.P.); 3 School of Medicine, Aristotle University of Thessaloniki, 54124 Thessaloniki, Greece; giannouliv@hotmail.com

**Keywords:** eating disorders, anorexia, bulimia, eating disorder voice, treatment

## Abstract

Background/Objectives: Individuals with eating disorders frequently describe encountering a highly critical internal voice that fixates on their eating habits, body shape, and weight. While existing literature acknowledges the significant impact of this eating disorder voice on affected individuals and its influence on treatment trajectories, research in this area remains limited. This study aimed to comprehensively examine and synthesize qualitative data concerning the experience of the eating disorder voice, with the goal of deepening our understanding of its fundamental characteristics and informing more effective approaches to assessment, treatment, and support in clinical settings. Methods: A systematic search was conducted across six databases for studies presenting qualitative findings relevant to the eating disorder voice. Fifteen studies were included, and their findings were reviewed and synthesized. Results: Results revealed that the eating disorder voice is often perceived by individuals as both protective and comforting, yet also controlling and intrusive, often seen as a force more powerful than themselves. Participants described a constant struggle to manage this internal criticism by differentiating themselves from the voice, with the fear of separation from the voice posing a significant challenge. Conclusions: This study underscores the complex nature of the EDV and its profound impact on individuals with eating disorders.

## 1. Introduction

Eating disorders (EDs) are prevalent and serious mental health conditions characterized by intricate biopsychosocial origins [1]. The term ‘EDs’ encompasses a spectrum of disordered eating practices, which have been increasingly recognized and defined in recent years. In essence, EDs represent profound mental health challenges marked by a persistent fixation on food, body weight, and shape [2]. Crucially, EDs are characterized by significant morbidity and mortality rates, as they are associated with a plethora of detrimental physical (e.g., malnutrition, obesity, etc.) and mental (e.g., anxiety disorders, obsessive-compulsive disorder, etc.) health outcomes, some of which can be life-threatening [3,4].

In the clinical literature of EDs, there are references highlighting a common occurrence wherein patients with EDs frequently deal with recurring and enduring thoughts concerning the necessity of adhering to strict regulations regarding food intake, as well as the control of weight and body shape [5]. This aspect is crucial for understanding EDs and can be approached through the concept of the ‘eating disorder voice’ (EDV). A substantial cohort of individuals diagnosed with an ED have reported experiencing an EDV [6]. This internal phenomenon often manifests as a hostile secondary or tertiary self-dialogue centered on eating habits, body image, and weight concerns [7,8]. Similar to pseudo-hallucinations [9], this voice is typically experienced as originating from within rather than being externally generated, although it may feel foreign to one’s own sense of identity. It is regarded as a distinct ‘sub-self’, encapsulating needs, emotions, perceptions, and behaviors detached from the individual’s overarching self-experience, despite its internally originated nature [10], with some theorists considering it a reflection of the ‘multi-voiced nature of human personality’ [6].

Literature findings highlighted that a significant portion of patients, ranging from 94% to 96%, frequently describe encountering an EDV [5,11]. Previous research has identified a pattern in how individuals perceive and interact with their EDV, suggesting a progression over time [6]. Initially, the EDV may be perceived as benign or supportive in the early stages of the ED, but it tends to become more hostile and controlling as the disorder progresses [12,13]. As the illness advances, this voice tends to be perceived as ‘malevolent’, ‘omnipotent’, and ‘powerful’ [11]. Coping with this inner voice is likened to a ‘battle’ and is linked with adverse emotions. The sense of entrapment and powerlessness in relation to this voice can complicate recovery efforts and potentially lead to relapse. In terms of recovery, existing literature indicates that gaining control over the EDV is crucial for overcoming disordered eating behaviors. As patients progress in their journey toward recovery, they often note alterations in the nature of this voice and their relationship with it [14]. More specifically, they describe a gradual acquisition of greater control over the voice [14].

Insights from cognitive models of auditory hallucinations shed light on this phenomenon [15,16], showing that the perceived power and intent of the voice significantly impact the individual’s experience. Research suggests that voices perceived as more powerful and malevolent tend to cause greater emotional distress, while those seen as benevolent are associated with lower distress levels and higher engagement [7,17,18]. In the context of disordered eating, the perceived power and malevolence of the EDV have been linked to more severe symptoms in anorexia nervosa (AN), including negative attitudes toward eating, lower body mass index, longer illness duration, and increased use of compensatory behaviors [7]. However, it is crucial to acknowledge that the existing literature predominantly examines the experience of the EDV within the AN population. Consequently, non-AN populations and individuals with other EDs are frequently neglected, depriving scholars and therapists of valuable insights into the similarities, overlaps, and differences in the manifestation of the EDV across the entire spectrum of EDs. Further, it is noteworthy that these cognitive models typically pertain to literal external voices observed in schizophrenia and schizoaffective disorders. While these models share a commonality with the experience of internal voices in EDs, their direct applicability to EDs may be constrained.

Various therapeutic modalities, including relational, dialogical, and narrative therapies, have been developed to address these detrimental beliefs and the subordinate, powerless attitudes individuals hold toward their negative thoughts, mindsets, or critical aspects of the self [19]. Similarly, therapeutic interventions, such as compassion-focused therapy, involve nurturing a “compassionate self/mind” to counterbalance a more destructive aspect of the self, namely, the “self-critic” [20]. In recent years, innovative therapies, such as Avatar therapy, have emerged to tackle the challenges posed by the self-critic and EDV [21]. Despite cumulative research and numerous therapeutic strategies, the efficacy and remission rates of current psychological, neurobiological, and pharmacological therapeutic approaches remain dissatisfactory [22,23]. Consequently, a universally endorsed treatment paradigm for EDs is currently lacking, even within the official practice guidelines of the American Psychiatric Association [24] and the British Psychological Society [25], given the variability in therapeutic interventions and distinct types of EDs. In light of this, it is prudent to assert that establishing a better understanding of EDs, with the ultimate goal of formulating efficacious and enduring therapeutic interventions, stands as a paramount objective.

While the existing literature acknowledges the significant impact of the EDV on individuals with EDs and its influence on treatment trajectories, research in this area remains limited, but is gradually expanding. However, a comprehensive review of qualitative studies specifically focusing on the experience of the EDV among individuals struggling with EDs is yet to be undertaken. This gap underscores the necessity for the present study, which seeks to deepen our understanding of the fundamental characteristics of the EDV and, in turn, inform more effective approaches to assessment, treatment, and support in clinical settings. By systematically gathering qualitative evidence from carefully selected studies adhering to rigorous methodological procedures, this study aims to contribute to the field by offering a novel conceptual perspective into the experience of the EDV and examining the following research questions: (1) What are the core characteristics and dimensions of the EDV, as experienced by individuals struggling with EDs? (2) How does the EDV influence the treatment trajectories and recovery experiences of individuals with EDs?

## 2. Materials and Methods

A qualitative meta-synthesis represents a scholarly investigation targeting the systematic interpretation and integration of findings derived from qualitative research reports. Meta-syntheses, as conceptualized by Thorne et al. [26], offer comprehensive amalgamations that yield novel insights from collective findings, surpassing individual contributions. The current synthesis aimed to consolidate, synthesize, and analyze qualitative narratives documenting the lived experiences and challenges of individuals with EDs concerning the EDV. The synthesis was conducted in accordance with the methodological guidelines proposed by Sandelowski and Barroso [27]. This methodological framework comprises the formulation of a precise research question, a systematic and exhaustive search of pertinent literature, the critical appraisal of identified studies utilizing a well-established quality assessment tool, the extraction of relevant data, and the application of a data analysis method. By adopting this framework, our objective was to provide a comprehensive and holistic understanding of the EDV phenomenon, transcending isolated perspectives.

### 2.1. Search Strategy

The meta-synthesis process involved a systematic review of qualitative studies, employing rigorous criteria for study selection and data extraction. We chose to focus exclusively on peer-reviewed journal articles to ensure consistency in research quality and methodological transparency. The search strategy utilized terms (‘eating disorders’ OR ‘anorexia nervosa’ OR ‘bulimia nervosa’ OR ‘binge eating disorder’ AND ‘eating disorder voice’ OR ‘anorexia voice’ OR ‘anorexic voice’) developed by the authors following an initial scoping search focused on qualitative studies related to EDs and the experience of the EDV. This tailored strategy was implemented across six databases (PubMed, PsycINFO, CINAHL, Embase, Medline, and Web of Science). Inclusion criteria comprised studies that (1) included participants with a current or former diagnosis of an ED, (2) focused on the EDV, (3) used qualitative methods of data collection and analysis, and (4) were published in English between 2005 and 2024. The language requirement was set to mitigate potential misinterpretations arising from cross-linguistic variations and/or unforeseen translation errors. Additionally, mixed-methods studies reporting qualitative data separately were considered eligible. Studies that did not abide by the inclusion criteria and were not published in peer-reviewed journals were excluded. The aforementioned established selection criteria ensured the appropriateness of the extracted data concerning the sample employed and methodological approaches.

### 2.2. Study Selection

**Figure 1:**
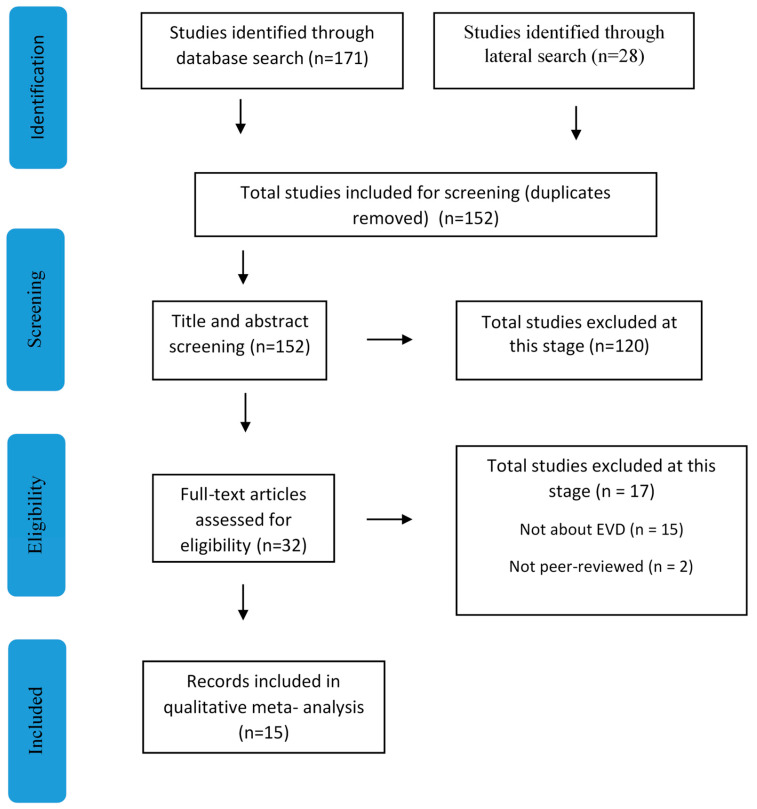
PRISMA flow diagram of the search process, study selection, and exclusion.

The search process yielded a total of 171 studies identified through database searches, supplemented by 28 studies from lateral searches (Figure 1). After eliminating duplicates, 152 studies remained for screening. Subsequently, the first author independently screened titles and abstracts, resulting in the exclusion of 120 studies during this phase. Exclusion criteria encompassed literature reviews, editorials, letters, conference abstracts, and unpublished dissertations. Studies lacking at least one illustrative quote delineating individuals’ experiences with the EDV were also excluded. Thereafter, 32 full-text articles were assessed for eligibility, leading to the exclusion of 17 studies, primarily due to irrelevance to the EDV (n = 15) and non-peer-reviewed status (n = 2). Ultimately, 16 records met the inclusion criteria and underwent rigorous content and quality assessments. Critical reflection was applied throughout the selection process to ensure transparency and mitigate potential biases. To manage the literature, we used a reference management tool to organize and track the articles found during the review process. Specifically, we employed Mendeley to store, organize, and manage the citations retrieved from electronic databases. This tool allowed for efficient citation management, enabling us to systematically track and retrieve relevant studies for further analysis.

### 2.3. Quality Assessment

To evaluate the quality of studies meeting the inclusion criteria, we utilized the Critical Appraisal Skills Programme (CASP) checklist due to its accessibility and widespread use in meta-syntheses, particularly in healthcare research [28,29]. We adopted a grading system adapted from Fox et al. [30], considering the updated CASP scoring (Critical Appraisal Skills Programme, 2018) since its publication. Papers scoring 8 or above were categorized as A, indicating a low likelihood of methodological flaws, while scores ranging from 4.5 to 7.5 were assigned a grade of B, denoting a moderate likelihood of methodological flaws. Papers scoring 4 and below were designated as C, suggesting a high likelihood of methodological flaws. The CASP scores and corresponding grades are summarized in Table 1. All papers underwent quality assessment by the first two authors. To enhance objectivity and rigor, approximately 25% (4 out of 14) of the papers were randomly selected for independent assessment by the third author. Agreement on 95% of scores was unanimous, and any remaining discrepancies were resolved through discussion among the authors. No studies were excluded based solely on low-quality ratings, aligning with the recommendations of Sandelowski and Barroso [27].

### 2.4. Data Extraction and Synthesis Procedure

The process of data extraction was conducted by the first three authors. Following the guidelines outlined in meta-synthesis literature [31], relevant information from the findings or results sections of the papers was extracted for synthesis. Consistent with established procedures for meta-synthesis in qualitative studies [27,32], our approach paralleled that described by Ludvigsen et al. [33], where we acted as ‘third-order interpreters’. We included quotes from participants (‘first-order interpreters’) and observations by authors (‘second-order interpreters’). However, in line with the recommendations of Sandelowski and Barroso [27], emphasis was placed on extracting quotes directly from the qualitative studies to highlight the first-order experiences of participants. Each study was comprehensively reviewed by the authors to ensure familiarity with the literature. Themes were developed cumulatively as the authors sequentially reviewed and extracted information from the studies, including existing quotations. This process was iterated a second time to refine the themes established in the initial extraction cycle. Findings from each study were extracted and entered into NVivo (QSR International, 2020), where they were categorized and summarized. Abstract summaries were created, and effect sizes were calculated when applicable. Regular consultations with the research team occurred throughout this process, and themes were deliberated with all authors until consensus was achieved.

Methodological and participant characteristics, such as the author(s) name, country, qualitative approach, and sample size, were extracted from each study and documented in Table 2. It is important to note that ethical approval was not required for this review. Finally, the Enhancing Transparency in Reporting the Synthesis of Qualitative Research (ENTREQ) statement was employed to enhance the reporting of the meta-synthesis (Appendix A).

## 3. Results

The analysis of the findings resulted in two main themes, each with two sub-themes. Table 3 below presents the themes and sub-themes along with their effect sizes.

### 3.1. Features of the EDV

Studies presented various features of the EDV, highlighting its dual nature. Some participants viewed it as protective and comforting, providing emotional support and guidance during difficult times. Others experienced it as controlling and intrusive, driving harmful behaviors and exacerbating feelings of guilt and distress. These differing perspectives are reflected in the following sub-themes.

#### 3.1.1. Protective and Comforting

One characteristic of the internal voice, as elucidated by several studies, is its role in providing protection and solace. For instance, in a study conducted by Ling et al. [34], numerous participants articulated that their EDV served as a protective mechanism against pain, vulnerability, and life challenges, imbuing them with a ‘sense of security’. Similarly, findings from Tierney and Fox [18] revealed that some participants perceived their internal voice as instrumental in decision-making amidst the “confusion, barriers, and messiness of life”. Furthermore, additional studies have highlighted participants’ perceptions of the internal voice as a companion that alleviates feelings of loneliness, fostering a sense of companionship and reducing isolation [18,19,20,21,22,23,24,25,26,27,28,29,30,31,32,33,34,35]. By assuaging negative emotions and ameliorating adversities through its constant presence, participants construed the internal voice as a guardian shielding them from detrimental consequences:

*“Despite all that you’ve taken from me, I’ve also come to realize that at other points in my life, you probably saved me. You gave me a voice when I lost mine and allowed me to cope, albeit in a destructive way, with extremely difficult situations”*

[35].

Other participants characterized the internal voice as akin to a trusted ‘friend’, with one likening it to a parental figure, offering comfort, guidance, and advice [12]. This attribute of support and reassurance was echoed by others, who depicted it as a guiding beacon promising personal enhancement and a sense of security when adhering to its directives [36,37,38]. Compliance with the voice’s dictates was perceived to mitigate negative affect and facilitate emotional regulation, shielding individuals from distressing emotions:

*“You get into this mind frame of, I don’t even think its yourself anymore, it’s just this other kind of thing or this other person that, erm, praises you when you encourage those harmful behaviors, so there are moments when your emotions, erm, are quite elated”*

[37].

#### 3.1.2. Controlling and Intrusive

Participants in many studies characterized the EDV as “intrusive,” “critical,” “harsh”, and “demanding”, perceiving it as a force more powerful than themselves [13,36,38]. In discussions of the anorexic voice, participants noted a transition marked by a cognitive shift that resulted in a division between the self and the anorexic voice. This experience was frequently described as a ‘battle between two minds or voices’ [13], the influence of which led women to disregard their rational thoughts and neglect their bodily needs, such as food: “something else inside me that would overtake me... it drives you to do the most insane things” [14]. The anorexic voice harshly condemned eating, inducing feelings of guilt whenever they did eat [14]. Healthcare professionals similarly noted that the EDV drives individuals to restrict caloric intake or over-exercise, leading to significant physical and psychological distress [39]. This voice was often described as possessing them, taking over their lives and identities [40]. The anorexic voice was particularly self-critical and became more dominant when participants attempted to recover or achieve ‘a sense of wholeness’:

*“The voice of anorexia constantly tells me, ‘You can’t do that, you shouldn’t do this, you’re not good enough, you’re not thin enough, no one will ever want you, you have no friends, you’re boring, you’re ‘damaged goods’, you have to exercise or you’re a failure”*

[13].

The voice was described as having a coercive nature, intensifying in volume and asserting a desire to ‘punish’ individuals for non-compliance with its dictates regarding food abstinence and lifestyle choices [12]. It was perceived as demanding sole allegiance, seeking to alienate individuals from familial, social, and professional support networks that sought to intervene in the disorder’s progression. By fostering antagonism toward such figures, the voice engendered a belief that individuals “don’t need them” or “don’t deserve them” [18]. Additionally, some participants compared the voice’s demanding nature to bullying, which was ‘overpowering’, rendering resistance futile [41]. Moreover, participants reported a perception that they were reliant on the voice for survival, thus exacerbating its demands and imposing unrealistic expectations. These expectations, perceived as extrinsic and unattainable, precipitated feelings of depression among participants:

*“It makes me depressed and suicidal because I am never good enough to meet my expectations, or rather the expectations of the voice in my head. (...) I wouldn’t be able to make any decisions because I forgot a long time ago what I wanted... now all my decisions are made to satisfy the voice in my head”*

[42].

### 3.2. Path to Healing and Restoration

Interestingly enough, this protective and comforting or conversely controlling and intrusive nature of the internal EDV extended to the individual’s path to healing and restoration. Ergo, to little surprise, the aforementioned dyadic dynamic between the individual and the EDV presents as a key aspect within the therapeutic context, albeit it appears to be rather paradoxical. This bidirectional relationship is explored in the following sub-themes.

#### 3.2.1. Managing the Internal Criticism

Studies revealed that the management of the internal critic in the context of recovering from the EDV reflects a dual perception: the voice is simultaneously perceived as an external alienated, non-psychotic entity, as well as an integrated aspect of the patient’s identity. Most notably, the differentiation between the self and the EDV as distinct entities is regarded to be profoundly pertinent within the psychotherapeutic framework across multiple interventions. Indeed, several studies highlighted the importance of externalization techniques designed to dissociate the individual’s identity, thoughts, and feelings from those of their EDV, fostering a detached meta-cognitive perspective. These techniques include, but are not limited to, the two-chair work (also known as voice dialogue) therapy and Avatar therapy.

For instance, several studies suggested that these vocalization and externalization techniques aid participants to be more proficient in categorizing their own thoughts and feelings compared to the ones generated from the EDV [40], allowing them to “vocalize the different thoughts” and offering them a “sense of relief” [34], by reducing “blame and frustration”, as the disorder is materialized as a distinct external entity they struggle against, separate from their personal identity, while still coming from within [38,39]. Therefore, a dyadic status emerges here, where the EDV is perceived as both an integral part of one’s identity, as well as an invasive external constituent that penetrates the patients’ personality and needs to be managed:

*“I need to acknowledge a very important part of my learning through the ED group: the very fact of addressing a letter to you means that I am acknowledging your existence. Yes, you exist as a distinct entity with your own unique voice—inside my head. You are part of my consciousness and mind—and yet you are separate. At least, I have been learning to separate your voice from my other voices and layers of my identity”*

[35].

Consequently, many participants identified less with the EDV and became more cognizant of their true situation to which they were oblivious before and used to consider “normal” or “just a bad habit” [40], due to the fact that they were “consumed” by the EDV [34]. Accordingly, separating the entity of EDV from their own self was described as allowing patients to “finally see pieces of truth” and helped them be more “self-forgiving”, “rational”, and “logical”. This was perceived as making them feel empowered enough to shift their perception of the information presented by the EDV from “reality” and “hard fact” to “unkind” and “nonsensical” by actively challenging it [14,35]. This process allowed them to start grasping the negative impact the disorder had on their lives and deconstructed the false promises of the perfect self by highlighting them as a “false sense of control” and a “false sense of security” [14,18,21,34]. As a result, this empowered the individuals to elevate from a passive to an active role, and the voice started losing its influence:

*“I wasn’t talking from a weaker point of view. I was talking at an equal’s kind of point of view and getting my point across, but also showing appreciation in a genuine way […] I feel like an equal status to it, rather than like a lower who’s being bullied. It’s like, ‘Okay, I’m here now, and we’re on the same level’”*

[34].

At the same time, this change in the dynamic in the relationship between the EDV and the individual was viewed by participants as allowing them to openly communicate their needs, such as asking for constructive feedback that motivates the individual toward “living a fuller life”, rather than spiteful “bludgeoning” criticism [35]. However, this process was viewed as evoking adaptive emotions, such as anger and desire for retaliation, and the voice in the studies was portrayed as an antagonistic presence, with participants claiming that “The voice feels like it’s this person that I want to get angry back at. Shout at, and just say: ‘Shut the fuck up, I don’t need you in my life’” [34]. One participant articulated this dual content poignantly:

*“Telling me I shouldn’t eat that chocolate bar because it has no nutritional value and is only harmful to my health is great. Telling me to eat it because I’m useless and have no hope anyway, and then telling me I’m useless and have no hope because I ate it... well to that I can now say, ‘fuck off.’ Your criticisms have created the conditions under which I have struggled, the constant questioning and hesitating, and then you have turned the blame back on me by convincing me it is only evidence of my lack. In this way, you have been entirely counterproductive. And again, to that I can now say, ‘FUCK OFF!’”*

[35].

#### 3.2.2. Fear of Separation

Understandably, for the majority of patients, the act of separating the self from the EDV was by no means a straightforward or unchallenged task. While a multitude of mechanisms were identified by participants to hinder this separation and, therefore, the subsequent path to healing and restoration, the most central barrier was described to be the fear of separation. More specifically, given the EDVs’ protective and comforting nature, which was previously examined in the antecedent theme, participants often described nurturing an “emotional attachment” toward the EDV. They reported feeling “scared to let go of” the EDV [40], with attempts to separate oftentimes being described as “killing your best friend” [12,18]. Accordingly, it became evident from findings that although participants expressed desires to overcome their ED, this process is also foregrounded by diffidence regarding their overall capacity to function independently. As noted by a series of studies, some individuals considered their EDV to be their “whole life” and would feel “lost” [18] or they “wouldn’t know how to live” [40] without it. Correspondingly, this sentiment was echoed by Ling et al. [34], where participants expressed a similar apprehension:

*“I just think [sniffles] as much as I don’t want it, it is my friend. Like I’m scared to let it go […] It’s made me feel like I don’t actually know how people cope with day-to-day life without having something like this to like rely on”.*

Interestingly, this perspective extends well beyond mere feelings of lost companionship. As mentioned earlier, adding to the sense of loss, it appears that patients also experience a sense of identification with the EDV, so much so that some patients felt that the latter had “taken over” their whole being and “stolen” their lives [12], viewing it as “sort of my identity […] this is who I am, this is all I know” [43], with others going so far as to claim that their “whole identity” is “nothing else” than their eating disorder and their weight [41]. Similarly, for some patients, this culminated to the extent where they feared they would be null without their EDV, stating that they “wouldn’t feel like a person at all” without it [41]. Hence, any attempts to separate the EDV were equivalent to “breaking apart a part of you” [41] and were perceived as “dissecting” by both the individual and their healthcare providers, who grew weary that excessive enforcement of this dissection might adulterate the individual, rendering them “fragmented” and make them “lose sight of who they are” [39]. Therefore, as it appears, patients viewed the EDV as an essential and structural constitutive of their selfhood, with its loss resulting in profound feelings of incompleteness:

*“I just have no idea what, what would happen, it feels like if someone said to you, well, what would you do if someone just came along and chopped your legs off, well you, I don’t know, I’d just flail around and it would just, I think it would just be very, it would be so much more distressing”*

[41].

Despite this, many patients viewed recovering from the disorder as more desirable than maintaining the EDV [14,18]. In order to successfully overcome the fear of separation, it was thought that participants are required to forge a new distinct identity untrammeled by the EDV [14,41]. In this transformative journey, patients are called to “reinvent” their personality in order to be “more free” to be themselves [41], and reclaim aspects of their identity that had been “stripped” [43] and overshadowed by the disorder:

*“I have a new identity, I am a student, a friend, I have social life, and I know that people I know now don’t see me as anorexic, I might have a history of that, but they see me as other things first”*

[14].

## 4. Discussion

This review enabled us to compile qualitative evidence on the experiences of the EDV from both individuals that have been diagnosed with an ED and from healthcare professionals that provide support to this specific clientele. Two descriptive themes, each with two sub-themes, were identified, highlighting the challenging and intense nature of the EDV. The synthesized findings revealed the intricate and paradoxical relationship individuals with EDs have with the EDV. The dual nature of the EDV—as both a source of comfort and a mechanism of control—highlights the complexity of EDs as not merely behavioral conditions but as deeply intertwined with identity and self-perception. The protective aspects of the EDV suggest that it serves as a coping strategy for managing emotional distress and a perceived lack of control. This aligns with theoretical frameworks that view EDs as maladaptive responses to internal conflicts and external pressures [44]. The EDV provides a sense of agency, albeit through harmful means. However, the controlling and punitive dimensions of the EDV contribute to the maintenance of the disorder by perpetuating negative self-beliefs and behaviors. The personification of the EDV as an external entity underscores the internal struggle individuals face, akin to an abusive relationship where the perpetrator and victim are within the same person.

In the majority of the studies, the voice was characterized by individuals as harsh and critical, demanding strict adherence to its rules and condemning any attempts to deviate from them. The EDV was experienced as criticizing the body, inducing guilt after eating, and urging individuals to restrict their calories further or engage in excessive exercise as a way to counterbalance the criticism. This demanding nature of the voice reflects the discourse of the ‘good anorectic’ [45], where individuals suffering from AN are expected to be super-compliant with the disorder’s ‘rules’. However, this compliance appears to be enforced by the EDV, which is perceived as taking over their identity and life, leaving no space for opportunities to diverge or self-reflect on the negative repercussions of such over-compliance.

The over-compliance to the dictates of the EDV can also be understood through a sense of fear. In our study, the EDV was perceived as punitive when individuals failed to discipline their bodies through calorie restriction and food abstinence. A recent study on pro-ED websites identified themes of punishment among users, with some participants aiming to ‘coach’ others through punishment to achieve thinness, while users expressed a willingness to be punished [46]. This suggests that the concept of punishment is prevalent in both offline and online discourses, indicating that the EDV may be a common experience among individuals with EDs. Similar to previous studies [11,13], participants in this meta-synthesis described the relationship with the EDV as being abusive, characterized by a dynamic in which the voice exerts control, manipulates and bullies individuals, and seeks to isolate them from their families. This coercive influence fosters the belief that they are self-sufficient and do not require support from others beyond the voice itself.

In the synthesized studies, the voice was characterized as overwhelmingly dominant, to the extent that participants often perceived it as having dual aspects within their minds, leading to a loss of personal identity. This entanglement with the voice complicated recovery, as individuals expressed a desire for recovery yet simultaneously feared relinquishing their attachment to the voice. This attachment reflects a deeper identification with the voice, reinforcing the role of the eating disorder as a core element of self-identity. Theories on EDs frequently emphasize this intricate connection between the disorder and self-identity, highlighting its central role in the persistence of the condition. Bruch [44] proposed that AN arises from difficulties in forming a stable sense of identity. Consequently, AN, and thereby the EDV, may become deeply intertwined with individuals’ self-perception, offering them a sense of self-worth, comfort, pride, and companionship. Losing connection with their pre-disorder identity may have strengthened their attachment to the EDV and reinforced their alignment with the anorexic self.

From a feminist perspective, EDs are often understood as behaviors that develop in response to cultural and societal expectations surrounding femininity and the female body [47,48]. In this context, women may perceive their bodies as representations of their identity, with calorie restriction serving as a form of resistance against these societal norms. This interpretation is supported by qualitative research, where individuals with AN reported that restricting their food intake was a way to negotiate their sense of self [49]. Consequently, the EDV may symbolize the broader struggle for identity negotiation in a society where expectations regarding roles, appearance, and body image are increasingly pervasive. As such, inhibiting the EDV and seeking treatment may feel terrifying for individuals that desire to construct their own identity. However, recovering from EDs involves confronting the anxiety associated with uncertainty and embarking on a journey to uncover one’s ‘true self’ by disentangling personal identity from the disorder [43]. This leads to the critical inquiry of whether healthcare providers should approach EDs and the EDV primarily as medical conditions or as identity-related issues.

This review presents several strengths, notably its compilation of qualitative data that captured the distinct experiences of individuals with EDs through their own accounts. By highlighting diverse perspectives and characteristics of the EDV, the paper offers a comprehensive understanding of this phenomenon. Another strength of this analysis is that it did not focus solely on studies of AN, despite the fact that the majority of EDV research centers on this specific ED. This broader inclusion aimed to provide a clearer understanding and suggest that the EDV may be present in other ED subtypes as well. This emphasis encourages scholars to consider the EDV across a wider spectrum of EDs, recognizing that EDV may be relevant to other subtypes that often receive less focus. Additionally, the use of the CASP quality assessment tool significantly enhanced the rigor of the review, allowing for a systematic evaluation of each study included in the meta-synthesis and ensuring that studies were categorized based on their quality scores. This meticulous assessment process, combined with peer briefing sessions and reflexive practices, helped to minimize potential biases and ensured a more objective analysis of the data.

However, the review is not without limitations. Despite the apparent centrality of the EDV in the experience of individuals with EDs and its crucial role in the trajectory of the disorder and recovery, the number of qualitative studies identified was limited. Additionally, the focus on predominantly female participants across the studies risks perpetuating the stereotype that EDs are primarily a female issue, potentially overlooking the experiences of male individuals, which may differ or exhibit unique characteristics. The lack of male representation is a significant gap, as it reinforces gender biases within the field. Additionally, there is a need to include a broader demographic range, encompassing not only male and female participants but also individuals from the LGBTQ+ community, to capture the full spectrum of experiences associated with EDs, especially given that ED symptomatology has increasingly been identified in this demographic group [50]. Furthermore, the studies included were predominantly conducted in Western contexts, such as the UK, USA, and Canada, thereby limiting the exploration of the EDV across different cultural backgrounds. Therefore, the findings of this meta-synthesis may not be generalizable to non-Western populations. Disorders such as AN and BN are often regarded as culture-bound syndromes [51,52], with the cultural context deeply influencing both the experience of the disorder and the meaning ascribed to healing. Gooldin’s [53] ethnographic work, for example, underscores how anorexia can embody culturally specific ideals of “heroic subjectivity” within certain sociocultural frameworks, revealing how the phenomenology of anorexia may differ in non-Western contexts. In line with this, an anthropological approach to studying the EDV would enhance understanding of the culturally specific ways in which the EDV is expressed, experienced, and interpreted. Future research should, therefore, prioritize cross-cultural examination of the EDV to capture the nuances in its expression and to better address culturally specific needs in treatment and support frameworks. By acknowledging these limitations, we recognize the need for more inclusive and culturally sensitive research to better understand the EDV.

## 5. Conclusions

In conclusion, this review synthesized valuable qualitative insights into the experiences of the EDV. The findings underscored the dual nature of the EDV, characterized by both comforting and punitive attributes, and its significant influence on identity, self-worth, and behavior. On the one hand, individuals may see the EDV as a source of security and comfort in facing life’s challenges, while on the other, they experience it as an oppressive force, enforcing strict compliance with damaging ED-related behaviors. This complex relationship highlights the intense personal and emotional struggle that individuals endure, complicating the process of recovery. The implications of these findings are critical for therapists.

First, the EDV should be understood not merely as a symptom but as a core element of an individual’s identity, reinforcing the need for more holistic treatment approaches that address identity formation and self-concept alongside traditional medical interventions. Therapists should employ strategies that empower individuals to separate their sense of self from the EDV while confronting the fear of losing the perceived safety it provides. Techniques such as externalization from narrative [54] and dialogical self-theory [55] can facilitate this differentiation, reducing the EDV’s dominance and fostering personal agency. However, it is essential to consider that, for many, the EDV may feel inseparable from their identity and sense of self, making separation challenging. Research on therapeutic dynamics suggests that framing the EDV as a distinct “other” (such as naming it “Ed”) can risk oversimplifying the patient’s experience, as some patients may feel their struggles are minimized or even invalidated by this approach [56]. Approaches such as Internal Family Systems therapy, which conceptualize the EDV as one component within a larger, multifaceted self, may offer an alternative path. This can allow for a gentler, more comprehensive separation, helping to mitigate the EDV’s dominance while respecting and preserving the patient’s complex sense of self. Additionally, therapists need to be mindful of the coercive and abusive dynamics of the EDV and consider approaches that validate patients’ experiences while encouraging a redefinition of self. This reinforces the importance of developing therapeutic strategies that empower individuals to separate their sense of self from the ED while confronting the fear of losing the perceived safety the disorder provides.

Further, the review suggested that therapists need to be mindful of the coercive and abusive dynamics of the EDV and consider approaches that validate patients’ experiences while gradually encouraging a redefinition of self. Considering that therapists may bring their own biases and concerns to the treatment of EDs [57,58], it is important for them to engage in ongoing reflection and training to avoid reinforcing stereotypes or misconceptions about EDs and the EDV. By developing a cohesive and positive self-identity, enhancing self-compassion, and strengthening social connections, therapists can help individuals disentangle their identities from the EDV. A deeper understanding of the EDV’s role in shaping identity and behavior will not only enhance therapeutic outcomes but also contribute to more compassionate, individualized care for those struggling with eating disorders.

## Figures and Tables

**Table 1:** Quality assessment using the Critical Appraisal Skills Programme (CASP) for qualitative research.

Study	1. Was There a Clear Statement of the Aims of the Research?	2. Is a Qualitative Methodology Appropriate?	3. Was the Research Design Appropriate to Address the Aims of the Research?	4. Was the Recruitment Strategy Appropriate to the Aims of the Research?	5. Was the Data Collected in a Way that Addressed the Research Issue?	6. Has the Relationship Between Researcher and Participants been Adequately Considered?	7. Have Ethical Issues been Taken into Consideration?	8. Was the Data Analysis Sufficiently Rigorous?	9. Is There a Clear Statement of Findings?	10. How Valuable is the Research?	Overall Score	Grade
1	1	0.5	0.5	1	1	0	0.5	1	0.5	1	7	B
2	1	1	1	1	1	0.5	1	1	1	1	9.5	A
3	1	1	1	1	1	0	0.5	1	1	1	8.5	A
4	1	1	1	1	1	0	0.5	1	1	1	8.5	A
5	1	1	0.5	0.5	0.5	0	0.5	1	1	1	7	B
6	1	1	1	1	1	0	0.5	1	1	1	8.5	A
7	1	1	0.5	1	0.5	0	0.5	1	1	1	7.5	B
8	1	1	0.5	0.5	0.5	0	0.5	1	1	1	7	B
9	1	1	0.5	1	0.5	0	0.5	1	1	1	7.5	B
10	1	1	1	1	1	0	0.5	1	1	1	8.5	A
11	1	1	1	1	1	0	0.5	1	1	1	8.5	A
12	1	1	1	1	1	0	0.5	1	1	1	8.5	A
13	1	1	0.5	1	1	0	0.5	1	1	1	8	A
14	1	1	0.5	0.5	0.5	0	0.5	1	1	1	7	B
15	1	1	1	1	1	0.5	0.5	1	1	1	9	A

**Table 2:** Summary of included papers.

Study	Country	Sample Size	Males in the Sample	Aim	Data Collection	Analysis
Tierney and Fox, 2011 [12]	UK	21 participants with experience of anorexia nervosa	0	Explore people’s experiences of living with an ‘anorexic’ voice	Poems, letters, and	Thematic analysis
					reflective narratives	
Williams and Reid., 2012 [13]	UK, USA, Canada, and Australia	14 participants with anorexia nervosa restricting subtype	2	Explore the lived experience of anorexia nervosa from the perspective of those who use pro-recovery websites for eating disorders	Online focus group or an e-interview	Interpretative phenomenological analysis
		or eating disorder not otherwise specified				
Jenkins and Ogden, 2011 [14]	UK	15 participants with anorexia nervosa that defined themselves as either recovered or in recovery	0	Explore how women make sense of their recovery from anorexia nervosa	Semi-structured interviews	Interpretative phenomenological analysis
Tierney and Fox, 2010 [18]	UK	21 participants with anorexia nervosa	0	Investigate experiences of	Poems, letters, and reflections/descriptive narratives	Thematic analysis
				and reflections on living with an anorexic voice		
Cardi et al., 2022 [21]	UK	39 participants (21 with anorexia nervosa and 18 in remission)	0	Assess the experience of the EDV in people with anorexia nervosa or in remission,	Open-ended questions	Thematic analysis
				and the feasibility of creating and interacting with a computerized representation (i.e., avatar) of this voice		
Ling et al., 2022 [34]	UK	9 participants receiving input for anorexia nervosa	0	Explore the experience and acceptability of voice dialogue amongst individuals	Semi-structured interviews	Interpretative phenomenological analysis
				with anorexia nervosa who experience an EDV		
Brennan et al., 2015 [35]	Canada	6 participants with an ED	0	Explore the perspectives	Feedback forms and letters	Thematic analysis
				of women with eating disorder diagnoses regarding their experiences of participating in an emotion-focused therapy group treatment for eating		
				disorders		
Morrison et al., 2022 [36]	UK	12 participants that had previously received a diagnosis	0	Explore the relationship between adverse	Semi-structured interviews	Grounded theory
		of anorexia nervosa		experiences in childhood and the development of the anorexia nervosa		
Watterson et al., 2023 [37]	New Zealand	18 participants with lived experience of an ED	0	Explore the experiences of women with a history	Semi-structured interviews	Structural narrative analysis
				of an ED		
Burnett-Stuart et al., 2024 [38]	UK	9	0	Generate new insights into the nature of AVs and disordered eating	Semi-structured interviews	Thematic analysis
Graham et al., 2019 [39]	UK	15 participants	1	Explore the perceptions of the anorexia voice	Semi-structured interviews	Thematic analysis
				among healthcare professionals (HCPs) in specialist eating disorder services		
Broussard, 2005 [40]	USA	13 actively bulimic participants	0	Interpret and understand bulimia nervosa as	Interviews and personal diaries	Heideggerian
				women experience it		phenomenological process
Williams et al., 2016 [41]	UK	11 participants with a lifetime	0	Explore the nature of the relationship between	Semi-structured interviews	Grounded theory
		history of anorexia nervosa		the self and the eating disorder in individuals with a lifetime history of anorexia nervosa		
Williams and Reid, 2010 [42]	USA, Canada, Spain, South Africa, Australia, New Zealand, Romania, and India	14 participants that used pro-anorexia websites	1	Investigate the experiences and understandings of those who	Online focus group and e-mail interviews	Interpretative phenomenological analysis
				wish to maintain their anorexia and look at how these understandings may		
				affect their treatment experiences		
Higbed and Fox., 2010 [43]	UK	13 participants in treatment for anorexia nervosa	Not stated	Explore illness perception in anorexia nervosa	Semi-structured interviews	Grounded theory

**Table 3:** Summary of themes and sub-themes.

Themes	Sub-Themes	Effect Size
Feature of the EDV	Protective and comforting	53%
	Controlling and intrusive	67%
Path to healing and restoration	Managing the internal criticism	53%
	Fear of separation	53%

## Data Availability

Not applicable.

## Appendix A

**Table A1:** The ENTREQ Checklist.

Item	Guide and Description	Reported on Page No.
Aim	State the research question the synthesis addresses	3
Synthesis methodology	Identify the synthesis methodology or theoretical framework that underpins the synthesis and describe the rationale for choice of methodology (e.g., meta-ethnography, thematic synthesis, critical interpretive synthesis, grounded theory synthesis, realist synthesis, meta-aggregation, meta-study, and framework synthesis).	3
Approach to searching	Indicate whether the search was pre-planned (comprehensive search strategies to seek all available studies) or iterative (to seek all available concepts until theoretical saturation is achieved).	3
Inclusion criteria	Specify the inclusion/exclusion criteria (e.g., in terms of population, language, year limits, type of publication, and study type).	3
Data sources	Describe the information sources used (e.g., electronic databases (Medline, Embase, CINAHL, PsycINFO, and Econlit), grey literature databases (digital thesis and policy reports), relevant organizational websites, experts, information specialists, generic web searches (Google Scholar), hand searching, and reference lists) and when the searches were conducted. Provide the rationale for using the data sources.	3
Electronic search strategy	Describe the literature search (e.g., provide electronic search strategies with population terms, clinical or health topic terms, experiential or social phenomena-related terms, filters for qualitative research, and search limits).	3–4
Study screening methods	Describe the process of study screening and sifting (e.g., title, abstract, and full text review, and the number of independent reviewers who screened the studies).	3–4
Study characteristics	Present the characteristics of the included studies (e.g., year of publication, country, population, number of participants, data collection, methodology, analysis, and research questions).	10–14
Study selection results	Identify the number of studies screened and provide reasons for study exclusion (e.g., for comprehensive searching, provide numbers of studies screened and reasons for exclusion indicated in a figure/flowchart; for iterative searching, describe reasons for study exclusion and inclusion based on modifications to the research question and/or contribution to theory development).	3–5
Rationale for appraisal	Describe the rationale and approach used to appraise the included studies or selected findings (e.g., assessment of conduct (validity and robustness), assessment of reporting (transparency), and assessment of content and utility of the findings).	5–6
Appraisal items	State the tools, frameworks, and criteria used to appraise the studies or selected findings (e.g., existing tools: CASP, QARI, COREQ, and Mays and Pope [25], or reviewer developed tools, and describe the domains assessed: research team, study design, data analysis and interpretations, and reporting).	5–6
Appraisal process	Indicate whether the appraisal was conducted independently by more than one reviewer and if consensus was required.	5–6
Appraisal results	Present results of the quality assessment and indicate which articles, if any, were weighted/excluded based on the assessment and provide the rationale.	7–8
Data extraction	Indicate which sections of the primary studies were analyzed and how the data were extracted from the primary studies (e.g., all text under the headings “results/conclusions” were extracted electronically and entered into a computer software).	9
Software	State the computer software used, if any.	9
Number of reviewers	Identify who was involved in coding and analysis.	9
Coding	Describe the process for coding of data (e.g., line-by-line coding to search for concepts).	9
Study comparison	Describe how comparisons were made within and across studies (e.g., subsequent studies were coded into pre-existing concepts, and new concepts were created when deemed necessary).	9
Derivation of themes	Explain whether the process of deriving the themes or constructs was inductive or deductive.	9
Quotations	Provide quotations from the primary studies to illustrate themes/constructs and identify whether the quotations were participant quotations or the author’s interpretation.	15–19
Synthesis output	Present rich, compelling, and useful results that go beyond a summary of the primary studies (e.g., new interpretation, models of evidence, conceptual models, analytical framework, and development of a new theory or construct).	15–22
